# CERVICAL “INFLAMM-AGING” AND GENITOURINARY SYNDROME OF MENOPAUSE: A GEROSCIENCE CASE-CONTROL STUDY

**DOI:** 10.1093/geroni/igad104.1439

**Published:** 2023-12-21

**Authors:** Christina Stennett, Patti Gravitt, Rebecca Brotman, Michelle Shardell

**Affiliations:** University of Maryland School of Medicine, Baltimore, Maryland, United States; Center for Global Health, National Institutes of Health, Rockville, Maryland, United States; University of Maryland School of Medicine, Baltimore, Maryland, United States; University of Maryland School of Medicine, Baltimore, Maryland, United States

## Abstract

The genitourinary syndrome of menopause (GSM) is a reproductive aging condition that describes signs and symptoms of the female reproductive tract associated with declining estrogen that women experience during menopause. Up to 84% of menopausal women experience signs and symptoms of GSM, including vaginal dryness and vulvovaginal pain. “Inflamm-aging” is a chronic aging-related elevation of proinflammatory markers that has been linked with multiple aging-related health conditions, but little geroscience research has examined cervical inflammation and GSM. To fill this gap, we carried out a case-control study (N=133) of cisgender women aged 35-60 years, where women with clinical signs of GSM (cases) were matched with up to 3 controls based on age and vaginal microbiota composition, which are known to track with reproductive stage. Participants’ cervical secretion samples were assayed to quantify immune marker concentrations. Samples with at least three out of seven predetermined proinflammatory cytokines and chemokines (IL-1ß, IL-8, IL-23, MIP-1α, MIP-1ß, IP-10, RANTES) in the highest quartile were classified as having a proinflammatory state. Of 133 participants, 35 (17/47 cases, 18/86 controls) had a proinflammatory state. A proinflammatory state was associated with higher odds of GSM in conditional logistic regression additionally adjusted for education and body mass index (aOR=2.39; 95% CI: 1.05-5.46). Findings demonstrate that participants with GSM signs had higher odds of a proinflammatory (“inflamm-aging”) state, independent of multiple characteristics, including vaginal microbiota. Additional geroscience research is needed to identify pathways connecting immune markers with GSM to discover interventions that may slow the pace of genitourinary aging.

